# Orthostatic intolerance as a potential contributor to prolonged fatigue and inconsistent performance in elite swimmers

**DOI:** 10.1186/s13102-022-00529-8

**Published:** 2022-07-23

**Authors:** Lindsay S. Petracek, Ella F. Eastin, Ian R. Rowe, Peter C. Rowe

**Affiliations:** Division of Adolescent/Young Adult Medicine, Department of Pediatrics, Johns Hopkins University School of Medicine Baltimore, 200 N Wolfe St, Room 2077, Baltimore, MD 21287 USA

**Keywords:** Athletic underperformance, Overtraining, Postural tachycardia syndrome, Orthostatic intolerance, Post-exertional malaise, Chronic fatigue syndrome, Neurally mediated hypotension

## Abstract

**Background:**

Athletic underperformance is characterized by fatigue and an inability to sustain a consistent exercise workload. We describe five elite swimmers with prolonged fatigue and athletic underperformance. Based on our work in myalgic encephalomyelitis /chronic fatigue syndrome, we focused on orthostatic intolerance as a possible contributor to symptoms.

**Methods:**

Participants were referred for evaluation of fatigue and underperformance to the Chronic Fatigue Clinic at the Johns Hopkins Children’s Center. All patients were evaluated for overtraining syndrome, as well as for features commonly seen in myalgic encephalomyelitis/chronic fatigue syndrome. The latter included joint hypermobility, orthostatic intolerance, and non-IgE mediated milk protein intolerance. Orthostatic intolerance was tested by performing a ten-minute passive standing test or a head-up tilt table test.

**Results:**

Orthostatic testing provoked fatigue and other symptoms in all five swimmers, two of whom met heart rate criteria for postural tachycardia syndrome. Treatment was individualized, primarily consisting of an increased intake of sodium chloride and fluids to address orthostasis. All patients experienced a relatively prompt improvement in fatigue and other orthostatic symptoms and were able to either return to their expected level of performance or improve their practice consistency.

**Conclusions:**

Orthostatic intolerance was an easily measured and treatable contributor to athletic underperformance in the five elite swimmers we describe. We suggest that passive standing tests or formal tilt table tests be incorporated into the clinical evaluation of athletes with fatigue and underperformance as well as into scientific studies of this topic. Recognition and treatment of orthostatic intolerance provides a new avenue for improving outcomes in underperforming athletes.

## Background

Athletic underperformance, sometimes referred to as overtraining syndrome, is characterized by fatigue and the inability to sustain a consistent exercise workload as compared to a given individual’s baseline performance [1–3]. While nutritional factors, hormonal imbalance, and autonomic abnormalities have been proposed as contributors to this phenomenon [4], no consistent biomarker for athletic underperformance or overtraining has been identified. Available treatments have primarily focused on stress reduction, optimization of nutrition, and adequate rest.

Orthostatic intolerance is a clinical disorder in which symptoms such as lightheadedness, fatigue, headaches, nausea, and cognitive difficulties are provoked by upright posture and ameliorated by recumbency [5–8]. These symptoms are thought to be due to a combination of suboptimal cerebral blood flow and an exaggerated sympatho-adrenal response when upright [5–8]. Common forms of orthostatic intolerance in adolescents and young adults include disorders with measurable heart rate and blood pressure abnormalities, such as postural tachycardia syndrome (POTS) [9, 10], in which elevations in norepinephrine are prominent during upright posture, and neurally mediated hypotension [11, 12], which often is associated with a rise in epinephrine during orthostatic stress. These conditions are not mutually exclusive and can occur in the same person. Individuals with orthostatic symptoms also can have a substantial reduction in cerebral blood flow despite a normal heart rate and blood pressure response to upright posture [13], and are characterized as having low orthostatic tolerance. In this group, the number of orthostatic symptoms correlates with the reductions in cerebral blood flow.

We describe a case series of five elite swimmers evaluated for chronic fatigue and decreased athletic performance. All five were unable to replicate their usual practice speed and workload from day to day. Based on discoveries about the importance of orthostatic intolerance to the pathophysiology of fatigue and post-exertional malaise (PEM) in individuals with myalgic encephalomyelitis/chronic fatigue syndrome (ME/CFS) [14–18], we had a higher index of suspicion for orthostatic intolerance as a treatable contributor to their abnormal fatigue and prolonged, inconsistent performance.

## Methods

For this case series, eligible participants were elite swimmers with symptoms of abnormal fatigue and athletic underperformance. All were or became members of the USA Swimming National Team and had been referred by their coaches or the USA Swimming medical staff to the Chronic Fatigue Clinic at the Johns Hopkins Children’s Center from 1995 to 2020. All individuals underwent a careful history and laboratory work-up for common causes of chronic fatigue by a physician with experience in the evaluation of ME/CFS and orthostatic intolerance (PCR). This included a complete blood count with differential white blood cell count, a comprehensive metabolic panel (electrolytes, creatinine, urea, total protein, albumin, calcium, aspartate aminotransferase, alanine aminotransferase, alkaline phosphatase, total bilirubin), free T4, thyroid stimulating hormone, c-reactive protein or erythrocyte sedimentation rate, vitamin B12, measures of iron stores, and a urinalysis. Other tests were obtained selectively based on the initial results of the basic testing panel. We asked the last two participants to complete a Beck Depression Inventory.

There is a well-recognized association between joint hypermobility and chronic fatigue, and between joint hypermobility and orthostatic intolerance [19–27]. As a result, we routinely assess joint hypermobility in our evaluation of individuals with fatigue. The physical examination therefore included the nine-point Beighton score, a commonly used and reliable measure of joint hypermobility [28]. Joint hypermobility was considered present if the Beighton score was four or higher. Due to the 2020 coronavirus pandemic travel restrictions, the initial evaluation of two of the five participants occurred by telemedicine and did not include a complete physical examination. Both had a Beighton score assigned during the telemedicine visit; one of these athletes was examined in-person at a later date.

There is a strong association between orthostatic intolerance and chronic fatigue [13, 14, 17, 18]. As such, all individuals presenting to our clinic with prolonged fatigue also are evaluated routinely for orthostatic intolerance. Three of the five had a ten-minute passive standing test in the clinic [29]. For the standing tests, we recorded heart rate and blood pressure at one-minute intervals (a) while the patient was supine for five minutes, (b) while the patient was standing upright and motionless for 10 min, and (c) while supine at the end of the standing phase for two minutes. While standing, the patient’s heels were 2 to 6 inches from the base of the wall, with the upper back against the wall. A fourth patient evaluated by telemedicine was instructed to conduct the same test assisted by a colleague at home using a wristband heart rate monitor; blood pressure measurements were not obtained. One individual had a head-up tilt table test prior to referral. During the passive standing tests, we instructed patients to minimize movements. We ascertained symptom severity at the completion of the supine phase, at one-to-two-minute intervals while standing, and during the post-test supine phase. Individuals reported symptoms on a 0–10 scale, with 0 meaning absence of the symptom and 10 being the worst severity imaginable. For individuals 12–19 years, the diagnosis of POTS required at least a 40 beat per minute (bpm) increase in heart rate between the lowest supine value and the peak while standing; a 30 bpm increase was required for those 20 and older [7]. The diagnosis of orthostatic intolerance was made if individuals had provocation of their presenting symptoms during the standing test, regardless of whether they also met criteria for POTS. Healthy individuals usually tolerate the 10-min standing test or tilt test without developing symptoms.

Based on prior observations in our clinic that 31% of patients with ME/CFS have evidence of a non-IgE mediated milk protein intolerance, we ascertained for the common upper gastrointestinal symptoms associated with milk protein intolerance, namely epigastric pain, early satiety, and gastroesophageal reflux, often associated with recurrent aphthous ulcers [30]. In those with symptoms suspicious for milk protein intolerance, a trial of eliminating milk protein from the diet was conducted immediately after clinical evaluation to ensure that continued exposure to milk protein was not interfering with assessing the response to other interventions.

To protect the privacy of the individual athletes, we report their ages at the time of evaluation as under 20 or greater than or equal to 20, not the precise age in years. For similar reasons, we elected not to report actual competition times. The Institutional Review Board of the Johns Hopkins Medical Institutes had waived informed consent for a retrospective study using data collected as part of routine care.

## Results

The age, sex, type of swimming, and presenting symptoms of the five underperforming swimmers are displayed in Table 1. After a thorough medical history, physical examination, and laboratory testing, alternative causes of underperformance were excluded. All had normal serum sodium levels as well as normal urea nitrogen and creatinine levels, indicative of normal hydration. Each had a self-reported daily oral fluid intake exceeding 2.5 L. Table 2 shows the results of their orthostatic testing. Table 3 shows their treatment and outcomes. In light of important individual differences in symptoms and severity, we have provided more detail in the following case descriptions.

**Table 1:** Symptoms at presentation in five underperforming swimmers

Patient	Age in years	Sex	Type of Swimming*	Presenting Symptoms					
				Fatigue	LH	PEM	Headache	Cognitive difficulties	Other
1	≥ 20	F	100–200 m stroke	x	x	x	x	x	Recurrent sinusitis Myalgias Inability to sit still
2	< 20	M	Distance freestyle	x	x	x			Chills Abdominal pain Cough Sweating
3	< 20	F	Distance freestyle	x	x		x		
4	≥ 20	M	100–200 m stroke	x			x		Dysthymia
5	≥ 20	F	Mid-distance	x	x	x		x	Moderate depression Heat intolerance Presyncope

LH, lightheadedness. PEM, post exertional malaise

*Stroke refers to backstroke, butterfly, or breaststroke. Mid-distance refers to races of 200–400 m

**Table 2:** Results of orthostatic testing

Patient	Lowest supine HR	Peak HR standing	Δ HR	Supine BP	BP at peak HR	Symptoms during test
1	50	78	28	123/66	119/76	Min 1: Lightheadedness Min 3: Acrocyanosis Min 6: Fatigue Min 8: Shaky legs, cognitive spaciness, increased fatigue Min 9: Pain in legs, knees, head, taking deep breaths
2	46	83	37	118/57	106/66	Min 1: Lightheadedness Min 2: Fatigue in legs Min 3: Heavy arms, acrocyanosis Min 4: Increased leg fatigue Min 6: Legs heavy and more fatigued Min 8: Paresthesias (arms and legs), fatigued overall Min 9: Hot, nauseated, presyncopal *Had to sit at minute 9
3	48	88	40	132/62	146/65	Supine: Fatigue 4–5/10, lightheadedness 0/10 Min 2: Lightheadedness, increased fatigue Min 8: Acrocyanosis
4	58	90	32	NA	NA	Min 1: Lightheadedness and nausea Min 3: Arm fatigue 2/10 Min 6: Headache Min: 8 Arm fatigue 8/10 Min 9: Hot sensation and hand paresthesias
5	42	70	28			Blurry vision Leg numbness Increased fatigue after test

**Table 3:** Treatment and response

Patient	Type of treatment						Response to treatment
	↑ salt intake		Oral rehydration supplements	Compression garments	SSRIs	Other	
	diet	tablets					
1	X		X	X	X	Cow’s milk protein restricted diet	Reduction in frequency of sinus infections Improved consistency with training Swim times returned to her expected competitive level Qualified for the Olympic trials before choosing to retire
2	X	X				Increased fluid intake Loratadine 10 mg daily Periodic IV normal saline	Resumed his usual pre-illness training volume No major training interruptions for the next 10 years
3	X	X		X		Increased fluid intake Periodic IV normal saline at times of exacerbation in symptoms	Improved consistency with training and competition performance for the next 6 years
4	X	X	X		X		Resolution of underperformance in practice and competitions Times returned to the expected level Consistent practice and performance at national and Olympic competitions
5	X	X	X		X	Hormonal intrauterine device Methylphenidate extended release 20 mg daily	Substantial improvement in mood, lightheadedness, energy, attention, and concentration Increased ability to train Elected to prepare for medical school rather than continue training

### Patient 1

An elite female swimmer was referred for evaluation of a five-year history of chronic fatigue, unrefreshing sleep, headaches, myalgias, lightheadedness, a “spacey” feeling, difficulties with attention and focusing, and monthly sinus infections. Her symptoms began at age 12, when she developed frequent sinusitis and upper respiratory illnesses that caused her to miss more training time than her peers. Despite these symptoms, she competed in the Olympics for the US several years later. Seven to eight months after the Olympic Games, however, she experienced a sudden increase in the frequency and severity of headache, neck pain, myalgia, sore throat, lightheadedness, and fatigue, persisting for the next three years.

In the two years before her evaluation, swimming practice remained inconsistent due to fatigue. She typically was able to train vigorously for two days. By the third day of practice, she had to stop early or do an easier workout. This was followed by one or two days of lighter training, after which she would be able to resume hard training briefly before repeating this pattern. Over this period of time, she was unable to replicate her usual racing times, and often had to withdraw from swimming competitions due to increased fatigue after the first race.

At the time of her consultation, her training schedule remained inconsistent. Headaches occurred weekly in association with nasal congestion and worsened fatigue, associated with muscle discomfort and weakness when the fatigue was worse. Triggers for her fatigue included standing or sitting upright for long periods of time, being in a hot tub for more than five minutes, major emotional stress, or any departure from her daily routine. She reported feeling spacey and unfocused on most days. She also had frequent epigastric pain, aphthous ulcers every couple of months, early satiety, and a family history of milk allergy.

**Fig. 1:**
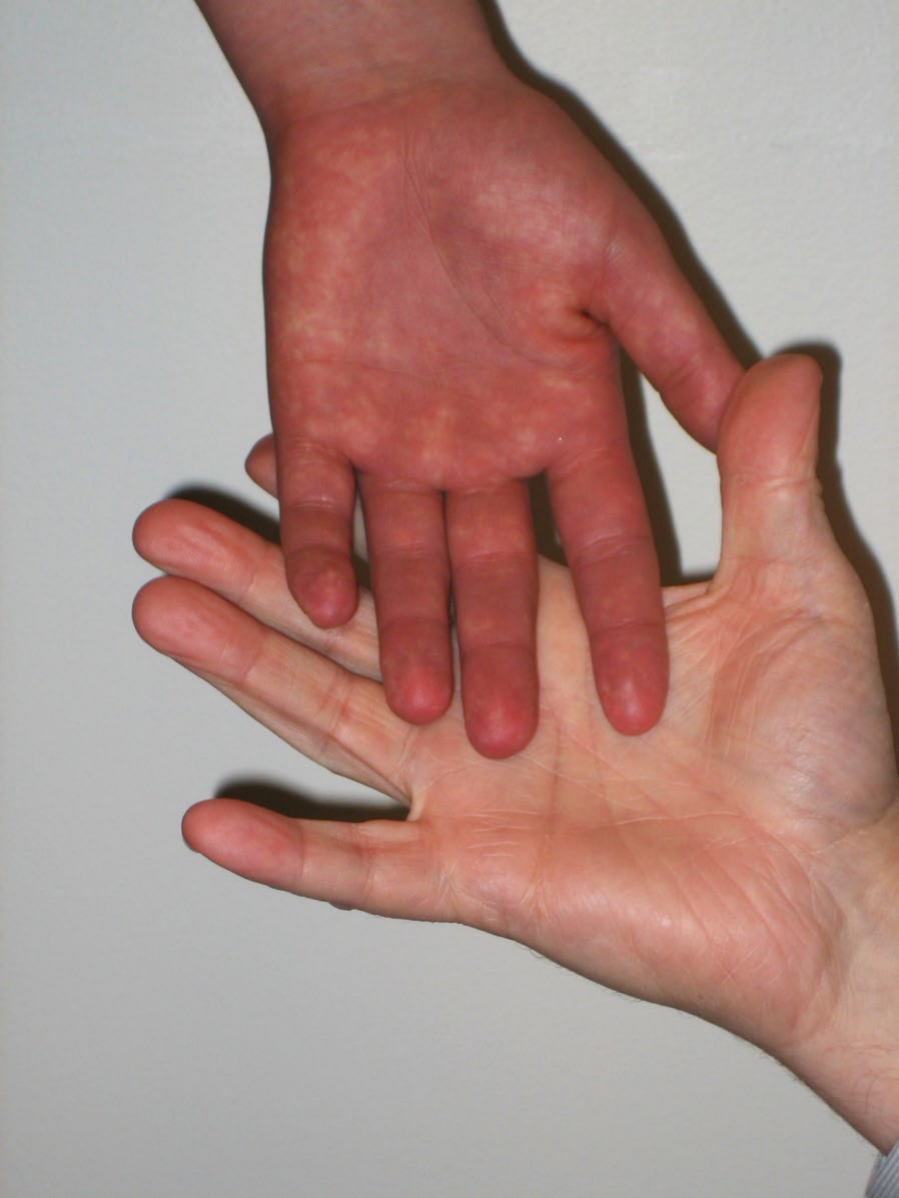
Acrocyanosis during a 10-min standing test. The examiner’s hand provides a color contrast

**Fig. 2:**
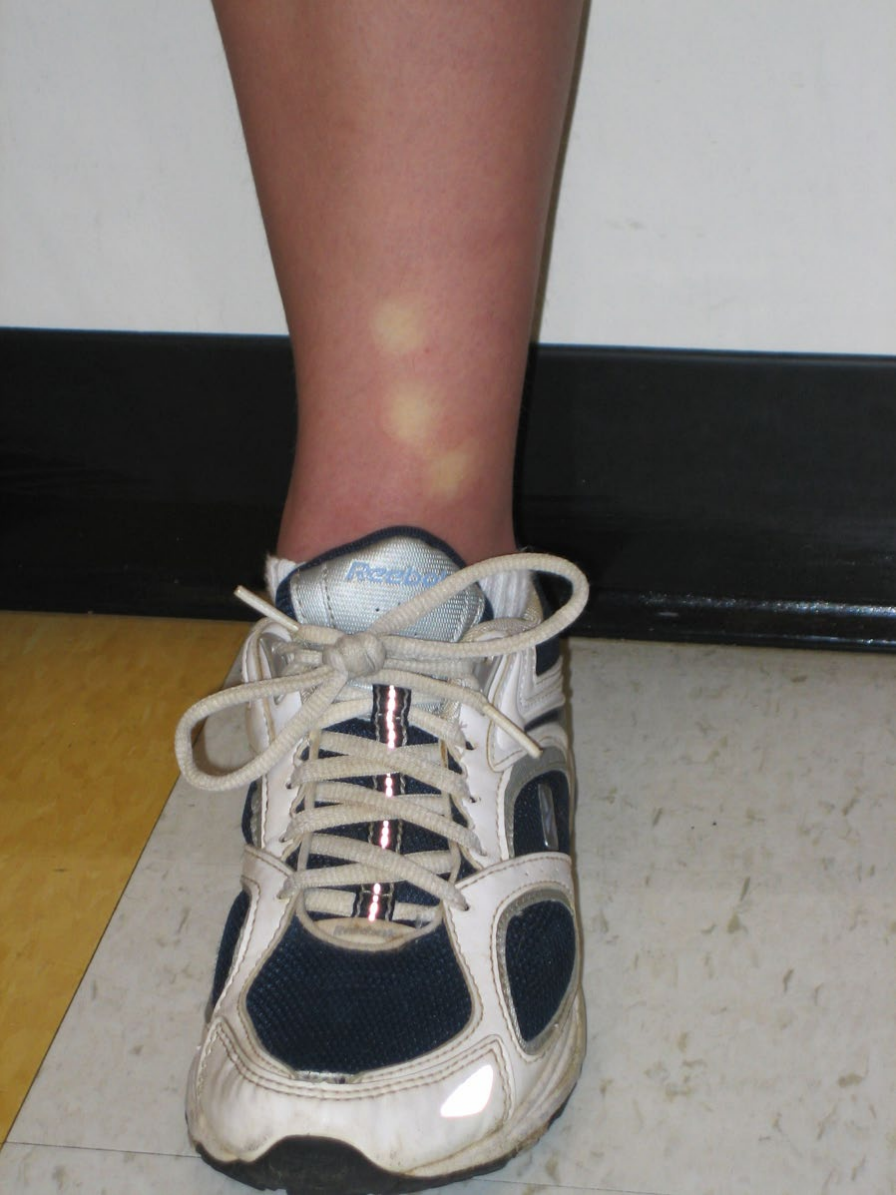
Delayed capillary refill during a 10-min standing test. The photograph was taken 6 s after digital compression of the lower limb skin

Her physical examination was notable for joint hypermobility (Beighton score 7/9). The passive standing test immediately provoked lightheadedness and at minute three she had a purple discoloration of the dependent limbs (termed acrocyanosis; Figs. 1, 2). By minute six, she reported increased fatigue, which worsened throughout the remainder of the test. By minute nine, she was taking deep breaths and had pain in her head, legs, and knees. Her 28 bpm change in heart rate did not meet the criteria for POTS, but the provocation of her typical symptoms and dyspnea while upright was consistent with orthostatic intolerance.

To address orthostatic intolerance, she was instructed to increase her sodium chloride intake according to taste. Her initial treatment also consisted of removing milk protein from the diet to address the upper gastrointestinal symptoms that were consistent with a non-IgE-mediated milk protein intolerance. Upon follow-up three weeks later, the sinus discomfort had resolved, and she reported improvement in fatigue. There was no change in her orthostatic intolerance symptoms; she therefore began a high sodium (90 mEq/L) rehydration drink, compression stockings when traveling, abdominal compression, and sertraline 25 mg daily (chosen because this was one of the few medications for orthostatic intolerance that was approved in competition; there was no self-reported or clinically suspected depression or anxiety). She experienced a prompt improvement in orthostatic symptoms and fatigue over the next two weeks. The consistency of her training improved substantially, returning to her pre-illness function, and becoming similar to the practice performance of her teammates. She was able to complete full competition days without excessive fatigue, and again qualified for Olympic Trials before electing to retire. The milk free diet was associated with an improvement in the frequency of her sinusitis episodes. Inadvertent or purposeful re-exposure to cow’s milk protein would lead to recurrences of the sinus discomfort.

### Patient 2

A male distance swimmer was referred for evaluation of a three-month history of fatigue and inconsistent performance. He had a history of lightheadedness when standing up after a long period of sitting.

His symptoms began at the time of a mild upper respiratory infection. During this time, his coach noted inconsistent performance during practices and frequent absences from practice. Other symptoms included abdominal pain on an intermittent basis, coughing, and episodes of sweating and chills after practice, typically followed by fatigue for the next 24 to 36 h. His sweating episodes usually occurred after attempts to increase the intensity and volume of his swim training, but also happened when standing for prolonged periods in warm environments.

He usually was able to perform well during swim competitions, but was much more fatigued afterwards compared to before the illness. Symptoms persisted despite decreasing his training from 7 to 9 sessions a week to 4–5 sessions and lowering his yardage from 10,000 to 6000–8000 yards per day.

His physical examination was notable for joint hypermobility (Beighton score 5/9). He also had allergic nasal inflammation. The history of fatigue and sweating prompted the passive standing test (Table 2). Upon standing, he had immediate provocation of lightheadedness. At minute two he began to feel fatigue in his legs, which became progressively worse throughout the remainder of the test. At minute nine, he suddenly became hot, nauseated, and presyncopal. The test was terminated prematurely at that point. At his age, the 37 bpm change in heart rate did not meet the criteria for POTS, but the provocation of his typical symptoms and presyncope while upright was consistent with orthostatic intolerance.

Treatment of orthostatic intolerance consisted of increased dietary intake of fluids and sodium chloride, as well as buffered sodium chloride tablets (2712 mg daily). For the nasal inflammation, he was treated with a non-sedating antihistamine (loratadine 10 mg daily). Within a month, he was able to resume his usual pre-illness training volume. He received 1–2 L of intravenous normal saline over 1–2 h periodically over the next several years as needed for exacerbations in symptoms outside of competitions. While continuing this regimen, he had no major interruptions in training over the ensuing decade as a member of the USA Swimming National Team and a university swim team.

### Patient 3

A female member of the USA Swimming National Team was referred for evaluation of a several month history of inconsistent training, fatigue, lightheadedness, and headache. Her fatigue was frequent, but worse after practice, in association with upper respiratory illnesses, and in warmer environments. Her headaches occurred primarily after standing up quickly and lasted for approximately 30–60 min. Her appetite had been lower as well. Lightheadedness was infrequent, occurring more commonly in warm environments.

Her performance at swimming competitions was not affected, but she felt an increased sense of effort and decreased strength during practice. Her only change in practice volume had been the addition of extra dryland sessions.

Upon evaluation, she had moderate joint hypermobility characterized by a Beighton score of 4/9. Her supine heart rate was 48 bpm and her peak standing heart rate was 88 bpm. Upon standing, she experienced lightheadedness, fatigue, and by minute eight she had moderate acrocyanosis. Her 40 bpm change in heart rate met the criteria for POTS.

Treatment consisted of increased fluid intake, two buffered sodium chloride tablets three times daily with meals (2712 mg daily), compression garments during air travel, and intravenous normal saline infusions of 2L over one hour as needed for episodic exacerbations in symptoms. We speculated that an inadequate sodium intake when she was at home contributed to the inconsistent practice performance, whereas a higher sodium intake in the prepared foods consumed at swim meets enabled the expected performance at competitions. After recognition and treatment of the orthostatic intolerance, she noted a prompt improvement within a month in all symptoms including her training consistency and performance at national and international competitions over the next six years.

### Patient 4

A male swimmer was evaluated via telemedicine for a six-to-seven-month history of athletic underperformance, abnormal fatigue between workouts, and headaches. His headaches had occurred frequently since high school, improving if he laid down. He had a history of fainting with medical procedures and venipunctures.

His fatigue first appeared approximately nine months before evaluation. Initially, it resolved after a two-week reduction in weight training, but significantly increased seven months before evaluation, temporally associated with a two-day period of intense training. Over the next seven months, he was unable to sustain his usual pace during practices. His weightlifting ability was not affected, but his performance in the pool was inconsistent. For example, in a practice set of 75 yard intervals of freestyle swimming, his pace in the first 75 yards would be adequate, but the second would be slower, and the third would be worse. He described the remainder of practice as “survival.”

His nutrition was reviewed by a dietician, who felt his caloric intake was adequate. A Beck Depression Inventory score was 11, consistent with minimal depression. His Beighton score was 7/9 during a telemedicine appointment. Due to the coronavirus pandemic travel restrictions, he was instructed to perform an in-home, supervised 10-min passive standing test. Upon standing, he had immediate provocation of lightheadedness and nausea. He developed arm fatigue by minute three which increased by minute eight, headache at minute six, and hand paresthesias as well as a hot sensation by minute nine. His 32 bpm change in heart rate together with the reproduction of his typical symptoms satisfied the criteria for POTS. His history of fainting with venipunctures was consistent with neurally mediated (vasovagal) hypotension.

Treatment consisted of an increased dietary intake of sodium chloride, supplemented by two buffered sodium chloride tablets three times daily with meals (2712 mg daily) and oral rehydration supplements that contained 2600 mg/L of sodium chloride. Within the first week of an increased intake of sodium chloride, he experienced a prompt improvement in the severity of symptoms. By the second week, he was practicing on a consistent basis, which represented a clear improvement. By week three, to address some persistent mild lightheadedness and fatigue, we initiated treatment with escitalopram (5 mg daily for four weeks then 10 mg daily thereafter). On this regimen he had resolution of the underperformance, both in practice and at national and Olympic competitions.

### Patient 5

A female swimmer (EFE) was referred for evaluation of athletic underperformance and associated presyncope, fatigue, unrefreshing sleep, heat intolerance, moderate depression, and brain fog. Due to the coronavirus pandemic, her evaluation was completed via telemedicine.

The onset of her symptoms began insidiously 15 months earlier at which time she began noticing heat intolerance and worse performance in competitions. Eight months before evaluation, immediately following two weeks of high-altitude swim training, she experienced a sudden episode of presyncope. Afterwards, she noted abnormal fatigue, and was unable to practice. She felt slightly better after a three week break from training. She resumed practice, performing well for three weeks, followed by resumption of symptoms. She had experienced mononucleosis nine months before the onset of symptoms, but recovered uneventfully from this illness.

At the time of her evaluation, she estimated that she could only manage 20–30% of the practice volume she previously tolerated, could not reach her previous intensity of effort during practice, and recovered in a delayed and incomplete manner. She would complete a normal practice but needed to nap afterwards, and experienced increased fatigue and difficulty with concentration for the next two days. She remained symptomatic the day after a practice and could not return to the pool until 48 h later. Her symptoms did not respond to another two-week break from practice.

On telemedicine examination, her Beighton score was 7/9. She had a head-up tilt table test at a 60-degree angle during which her supine heart rate was 42 bpm and her peak upright heart rate during the first ten minutes was 70 bpm. During the test she reported blurry vision and leg numbness, as well as increased fatigue afterwards. The test was consistent with low orthostatic tolerance. Her Beck Depression Inventory score was 19, consistent with moderate depression. An in-person evaluation eight months later confirmed the Beighton score, and excluded other examination abnormalities that could have been associated with fatigue.

Treatment included increased dietary sodium chloride intake, supplemented by two salt tablets three times daily (3810 mg daily), and oral rehydration supplements with a sodium chloride concentration of 500 mg/L. She underwent placement of a Kyleena intrauterine device for the increased fatigue and orthostatic intolerance she experienced around the time of her menstrual periods. For depression, she was treated with escitalopram, gradually increasing to an optimal dose of 25 mg daily.

Three months later, her mood, lightheadedness, and energy were greatly improved. She was not back to training vigorously but was improving each month and tolerating an hour of exercise each morning. At that time, she also had flushing and erythema consistent with allergies or increased histamine. Two months later, her mood was back to normal, and she continued to tolerate 60–90 min of exercise each day. However, she still would get several hours of post-exertional malaise and was unable to train at a competitive level. Methylphenidate extended release 20 mg daily led to a further improvement in attention and concentration, lightheadedness, and stamina. She elected to prepare for medical school admission rather than to continue training.

## Discussion

This case series draws attention to the potential for orthostatic intolerance to be an important contributor to the pathophysiology of inconsistent performance in elite swimmers. All five athletes reported fatigue as a consistent symptom, often worse in positions of upright posture or in hot environments, usually associated with lightheadedness. Testing that involved brief periods of upright posture provoked their typical symptoms, sometimes associated with orthostatic tachycardia. Individualized treatment directed at the orthostatic intolerance and other symptoms was associated with improvement, and in most cases resolution, of the inconsistency in training and competition performance. Larger studies will be needed to measure the prevalence of orthostatic intolerance among athletes with prolonged underperformance at all levels of competitive swimming, as well as in other sports.

Our case series suggests that attention to circulatory abnormalities may be informative in future studies of athletic underperformance or overtraining syndrome (OTS). Our observations are consistent with the findings of several other studies on OTS that have identified abnormalities in adrenergic tone, heart rate variability, and responses to head-up tilt or lower body negative pressure (another technique for measuring orthostatic intolerance) [1, 31–35]. These studies have tended to focus on autonomic problems as a consequence and marker of overtraining, rather than on orthostatic intolerance as a treatable, causal factor.

Overtraining syndrome is marked by underperformance, chronic fatigue, recurrent infections, and altered mood states such as depression and lack of motivation [36]. Athletes with high workloads, such as swimmers and endurance athletes, are especially prone to overtraining syndrome. No consistent biomarker in the blood has been identified [4, 37]. Recommended evaluations include an extensive search for hormonal abnormalities, metabolic and nutritional problems, psychological disorders, systemic infections such as mononucleosis, and dysfunction in any organ system. Treatments for OTS have included rest, dietary alterations to meet caloric needs and address vitamin deficiencies, and non-pharmacologic treatments such as massage [36]. Thus far, testing for and treatment of orthostatic intolerance has not been incorporated as part of the routine evaluation of prolonged underperformance. Moreover, little attention has been given to sodium chloride as part of the dietary approach. It would be important for coaches and training staff to be aware of the symptoms of orthostatic intolerance in underperforming athletes. However, we would emphasize that the evaluation and treatment of orthostatic intolerance needs to be performed by qualified medical personnel as part of a comprehensive evaluation of athletes with prolonged underperformance and abnormal fatigue.

The three main pathophysiologic features predisposing to orthostatic intolerance are reduced vasoconstriction/increased pooling of blood in the lower half of the body, low blood volume, and exaggerated sympathetic nervous system and catecholamine responses to upright posture [38]. These abnormalities can begin insidiously or can be triggered by a variety of infections, trauma, autoimmune responses, or other factors [6]. Orthostatic intolerance is more common in women and in individuals with joint hypermobility [19–27]. Treatment of orthostatic intolerance consists of non-pharmacologic interventions such as increased dietary sodium intake, adequate fluid intake, graded aerobic exercise, and compression garments such as compression stockings and abdominal binders [39–42]. When these measures are not sufficient, various medications are available to address suboptimal vasoconstriction, increased pooling of blood, low blood volume, and the exaggerated sympatho-adrenal response. Selection of the medications is individualized and often depends on whether a given medication can treat other comorbid problems. For example, both orthostatic intolerance and co-morbid anxiety/depression would be expected to respond to serotonin reuptake inhibitors. Table 4 lists the medications that could be allowed either with or without a therapeutic use exemption by the athletic anti-doping agencies for treating orthostatic intolerance in swimmers (from https://globaldro.com/US/search).

**Table 4:** Medications for orthostatic intolerance and their permissibility in competitive swimming

Medication	Comments	Allowed in swimming?
Vasoconstrictors		
Midodrine	Suggested as first line therapy for those with baseline hypotension	Yes, outside of competition; in competition requires a TUE
*Stimulants* Methylphenidate, dextroamphetamine, and others	Suggested as first line therapy for those with prominent cognitive dysfunction or a personal or family history of attention deficit hyperactivity disorder	Yes, outside of competition; in competition requires a TUE
Volume expanders		
Sodium chloride	Oral supplements not always sufficient as the only therapy. IV normal saline is impractical over the longer term, but can help restore baseline function after acute infections or as rescue therapy	Yes for oral sodium For IV fluids > 100 mL, a TUE is required
Fludrocortisone	Suggested as first line therapy for those with baseline hypotension or increased salt appetite. Potassium supplementation is needed due to increased urinary potassium excretion. Can aggravate acne	Yes, outside of competition; in competition requires a TUE
Hormonal contraceptives	Indicated for females with dysmenorrhea or when fatigue and lightheadedness worsen with menses	Yes
Desmopressin acetate	Suggested for those with nocturia. Hyponatremia can occur	No; requires TUE in and out of competition
Sympathetic tone and heart rate modifiers		
*Beta adrenergic antagonists* Atenolol, propranolol	Suggested as first line therapy for those with a relatively elevated resting heart rate, anxiety, or headache. Can exacerbate asthma. Contraindicated for diabetics	Yes
Pyridostigmine bromide	Effective in POTS and neurally mediated hypotension. Also helpful for GI motility problems	Yes
Clonidine	Suggested for those with anxiety, problems with attention, or insomnia	Yes
Ivabradine	Suggested for those with elevated baseline heart rate	Yes
SSRI/SNRI		
Escitalopram, sertraline	Indicated for dysthymia, depression, or anxiety	Yes
Duloxetine	Useful if myalgias are prominent	Yes

TUE, therapeutic use exemption; BPM, beats per minute; POTS, postural tachycardia syndrome; GI, gastrointestinal; SSRI, selective serotonin reuptake inhibitor; SNRI, serotonin norepinephrine reuptake inhibitor

Several factors could have influenced the expression of orthostatic intolerance in our series. First, joint hypermobility is common in swimmers (all five in our case series had a Beighton score of 4 or higher) and may confer a biomechanical advantage in swimming [43]. However, because the same connective tissue that allows for greater laxity in ligaments is also present in the wall of blood vessels, this predisposes to greater vascular compliance with venous pressure changes, such as increased hydrostatic pressure with standing [19, 44]. Upright posture therefore would be associated with greater pooling of blood in the dependent circulation, as has been confirmed recently in adults with ME/CFS and joint hypermobility [26]. Some dependent pooling can be countered by the use of compression garments. Racing swimsuits that provide increased compression in the abdomen and the lower half of the body might allow for better circulation and consequently better performance in competitions than during practice.

### Limitations

This case series establishes the concept that orthostatic intolerance can be a contributor to athletic underperformance. Due to the coronavirus pandemic restrictions, patient four did not have blood pressure recordings during the passive standing test, which might have confirmed neurally mediated hypotension. However, ten minutes is not always a sufficient amount of time to detect neurally mediated hypotension, and his history of fainting was consistent with the diagnosis and does not change his diagnosis of POTS. Patient five was also evaluated via telemedicine due to the pandemic, and we do not have her blood pressure recordings or minute by minute symptoms for similar reasons as well as the fact that she had a tilt table test performed at another institution.

As this was a case series, we did not record practice times, practice attendance, or hours of practice per week in a systematic manner. We did not ascertain whether post-treatment self-reported clinical improvements were accompanied by hemodynamic and symptomatic improvements in response to the same orthostatic stress. Nor did we use validated fatigue questionnaires at baseline and during follow-up. These details would be helpful to record in prospective studies. Disentangling the independent effects of serotonin reuptake inhibitors and sodium supplementation may be challenging in future studies; both interventions can have a beneficial effect on orthostatic intolerance. Conversely, simply feeling better and being able to practice more consistently after treating orthostatic intolerance (without anti-depressants) could have a beneficial effect on mood.

Additionally, since all participants were elite swimmers at the national or Olympic level, exact ages, competition times, and type of stroke were omitted to ensure anonymity. This was a relatively small, single center sample, so larger studies will need to be performed to confirm that our findings are applicable to other underperforming or overtrained athletes.

## Conclusions

This small case series is a proof of concept that orthostatic intolerance can be a treatable contributor to athletic underperformance. The mechanism by which orthostatic intolerance causes fatigue is not known. Fatigue and exercise intolerance are prominent symptoms in most forms of orthostatic intolerance [14], and these symptoms generally improve when a successful treatment of orthostatic intolerance is identified [18]. Further studies are needed to determine the optimal methods of orthostatic testing (passive or active standing tests versus prolonged head-up tilt testing). Orthostatic testing needs to be performed with medical supervision because of the potential for syncope and injury to occur. Whether prior orthostatic intolerance—as reflected in prior syncope, frequent lightheadedness, or excessive tachycardia in response to standing—will prove to be a risk factor for subsequent periods of prolonged underperformance remains to be determined. Randomized trials could assess whether sodium supplementation reduces the incidence of overtraining, and whether intermittent intravenous saline infusions could be effective in the early phase of underperformance to prevent further deterioration. Other studies could evaluate whether treating orthostatic intolerance accelerates the return of consistent practice performance in underperforming or overtrained athletes compared to prolonged rest and other currently recommended techniques.

## Data Availability

All data used in this study are included in the published article.
